# Lateral hypothalamic orexinergic neurons as central mediators of pain modulation and non-pharmacological analgesia

**DOI:** 10.3389/fphar.2026.1865248

**Published:** 2026-07-01

**Authors:** Yunhe Zhu, Shan Wang, Jingping Sun, Qidong Zhang, Guobi Chai, Qingzhao Shi, Jian Mao, Wu Fan, Jianping Xie

**Affiliations:** 1 College of Chemistry, Zhengzhou University, Zhengzhou, Henan, China; 2 Zhengzhou Tobacco Research Institute of CNTC, Zhengzhou, Henan, China; 3 Beijing Life Science Academy, Beijing, China

**Keywords:** lateral hypothalamus, neural circuit, non-pharmacological analgesia, orexinergic neuron, pain

## Abstract

Pain is a complex sensory and affective experience regulated by distributed neural circuits that integrate internal physiological states with external stimuli. Orexinergic neurons, a widely projecting neuronal population within the lateral hypothalamus (LH), play a central role in this process. This review synthesizes current evidence on the involvement of LH orexinergic neurons in nociceptive regulation and highlights their role as a key integrative substrate for non-pharmacological analgesia. Orexin peptides (orexin A and orexin B) modulate both the sensory-discriminative and affective-motivational components of pain via their receptors (OX1R and OX2R) in a context-dependent manner, producing antinociceptive or pro-nociceptive effects depending on peptide subtype, receptor distribution, circuit architecture, and pain modality. In parallel, emerging evidence indicates that orexinergic neurons are critically engaged in diverse non-pharmacological analgesic paradigms, including stress-induced analgesia, olfactory modulation, electroacupuncture, and exercise-induced hypoalgesia. In addition, other neuronal populations within the LH, such as glutamatergic, GABAergic, and neurotensinergic neurons, also contribute to pain regulation in a circuit-specific manner and partially overlap anatomically and functionally with orexinergic neurons. Collectively, these findings position LH orexinergic neurons as a central node linking neural circuit dynamics with the behavioral and physiological modulation of pain. Targeting orexin-related pathways may therefore provide novel avenues for the development of non-pharmacological and integrative pain management strategies.

## 1 Introduction

Pain is an essential sensory and emotional experience that serves as a biological warning signal that helps protect organisms from potential harm. While acute pain is widely regarded as an adaptive response, chronic pain represents a maladaptive state that affects nearly 20% of the global population, thereby imposing a substantial socioeconomic and health burden (Chen et al., 2022). The perception and regulation of pain depend on the dynamic interactions between ascending nociceptive pathways and descending modulatory systems within the central nervous system. The identification of specific neuronal populations that integrate diverse internal and external signals to modulate pain has become a central objective in pain research.

Among the brain regions implicated in pain regulation, the hypothalamus has emerged as a critical node linking nociceptive processing to physiological state and adaptive behaviors (Fong et al., 2023; Maroso and Stern, 2023). In particular, the lateral hypothalamus (LH) occupies a strategic position within this network owning to its extensive connectivity with both ascending nociceptive pathways and descending modulatory systems (Tracey and Mantyh, 2007). Somayeh et al. demonstrated that microinjection of carbachol into the LH reduced nociceptive behaviors, such as paw lifting, licking, and biting, in a dose-dependent manner in Wistar rats following formalin injection (Somayeh et al., 2015). Within the LH, orexinergic neurons constitute a discrete and functionally specialized neuronal population. Although fewer than 5,000 orexinergic neurons are present in the rodent brain, the extensive anatomical distribution of their fibers (Soya and Sakurai, 2020). These neurons innervate key regions involved in nociceptive transmission and modulation, including the spinal cord, periaqueductal gray, thalamus, amygdala, and cortical areas (Bibi Marjan and Hossein, 2017). Accumulating evidence indicates that orexinergic modulation of pain depends on pain modality, orexin peptide subtype, receptor distribution, sex, and circuit context.

In addition to their involvement in general nociceptive regulation, recent studies have increasingly implicated the orexinergic system in mediating the analgesic effects of a range of non-pharmacological interventions. Orexinergic neurons are known to regulate fundamental processes such as wakefulness, stress adaptation, reward processing, and metabolic homeostasis (Wang et al., 2022). These functions are closely intertwined with pain perception, suggesting that orexinergic neurons may serve as an integrative substrate through which internal state influences nociceptive processing. Approaches such as stress-induced analgesia, sensory modulation, electroacupuncture, and exercise-induced hypoalgesia have been shown to engage central circuits that overlap with orexinergic pathways (Dmitry et al., 2011; Kami et al., 2018). Although the mechanisms underlying these interventions are heterogeneous, a convergent feature is the recruitment of brain systems that integrate arousal, emotional state, and physiological context. In this context, LH orexinergic neurons may represent a common cellular substrate through which diverse non-pharmacological strategies exert modulatory effects on pain.

In this review, we summarize current evidence supporting the role of the orexinergic system as a central hub in pain modulation, with particular emphasis on the distinct functions of its neuronal populations in regulating both nociception and non-pharmacological analgesia. By synthesizing findings from anatomical, electrophysiological, and behavioral studies, we aim to elucidate how orexinergic circuits contribute to the central regulation and to highlight their potential for the development of novel analgesic strategies beyond conventional pharmacological approaches.

## 2 Orexinergic neurons and their downstream circuits in pain modulation

### 2.1 Orexinergic neurons as a core cellular substrate for pain regulation

Orexinergic neurons constitute a key neuronal population within the LH and perifornical area, primarily responsible for the synthesis and release of orexins also known as hypocretins (Chen et al., 2015). Orexins consist of two isoforms-orexin A (OXA) and orexin B (OXB)-which are derived from a common precursor peptide. These neuropeptides exert their physiological effects through 2 G protein-coupled receptors: orexin one receptor (OX1R) and orexin two receptor (OX2R). OXA binds with similar affinity to both receptors, whereas OXB exhibits approximately tenfold greater affinity for OX2R than for OX1R (Sakurai, 2014). Despite their relatively limited number, these neurons, through extensive projections to pain-related regions-such as the PAG, ventral tegmental area (VTA), nucleus accumbens (NAc), and spinal cord-coordinate nociceptive processing and pain-related behaviors in acute and chronic neuropathic pain models (Table 1).

**TABLE 1:** Orexinergic circuit modulation across pain modalities.

Pain category	Pain model	Subject	Behavioral assays	Key findings	Orexin-regulated pain dimension	References
Acute pain	Naïve mouse	Male C57BL/6, ORX-abl mice, ORX-tTA mice	Intradermal capsaicin injection into the cheek	1. LH orexin neurons oppositely regulated pain and itch 2. LH-PAG orexinergic circuit suppressed pain but enhanced itch 3. Orexin neurons involved in chronic itch processing 4. Potential target for persistent pruritus	Chemical nociception	Kaneko et al. (2024)
Acute pain	Naïve rat	Male wistar rats	Cataleptic immobility, tonic immobility, tail-flick test	1. OXA/OXB differentially regulated immobility responses 2. i.c.v. Orexins failed to induce tail-flick analgesia 3. VlPAG orexin B produced significant analgesic effect 4. Orexin-related analgesia linked to defensive response circuitry	Thermal pain, defensive response-related analgesia	Miranda-Páez et al. (2014)
Acute pain	Naïve rat	SD rats	Tail-flick test	1. OXA reduced tail-flick nociceptive responses 2. OXA decreased RVM on-cell activity and increased RVM off-cell activity 3. Descending pain modulation involved	Thermal pain	Azhdari-Zarmehri et al. (2014)
Acute pain	Naïve rat	Male wistar rats	Tail-flick test	1. Intra-LH carbachol induced tail-flick antinociception 2. VTA and NAc OX1R blockade reduced LH-induced analgesia 3. VTA OX1R showed a stronger contribution than NAc OX1R	Thermal pain	Sara et al. (2013)
Acute pain	Naïve rat	Male wistar rats	Hot-plate test	1. OXA produced vlPAG-mediated thermal antinociception 2. OX1R-dependent suppression of GABAergic inhibition 3. CB1/endocannabinoid signaling involved 4. Disinhibition of vlPAG neurons enhanced analgesic output	Thermal pain	Ho et al. (2011)
Chronic neuropathic pain	CCI model	SD rats	Von frey test, hargreaves test	1. Decreased spinal OX2R after CCI 2. Orexin B relieved CCI-induced mechanical and thermal hypersensitivity 3. Reduced spinal inflammation, oxidative stress and JNK/NF-κB signaling	Thermal pain, mechanical pain	Zhu et al. (2024)
Chronic neuropathic pain	CCI model	Female SD rats	Hargreaves test	1. LH carbachol stimulation reduced thermal hyperalgesia in CCI rats 2. Intrathecal OX1R blockade attenuated LH-induced analgesia 3. LH–spinal dorsal horn OXA/OX1R pathway involved	Thermal pain	Wardach et al. (2016)
Chronic neuropathic pain	CCI model	Male wistar rats	Von frey test, hargreaves test	1. Intra-LH carbachol reduced CCI-induced mechanical and thermal hypersensitivity 2. Effects showed dose-dependent tendency 3. OX1R and OX2R blockade reversed LH-induced analgesia 4. LH–spinal orexin receptor pathway involved	Thermal pain, mechanical pain	Sakineh et al. (2020)
Chronic pain	Migraine (trigeminal nociceptive)	Male SD rats	Von frey test, response of A-fibre and the C-fibre	1. OXA inhibited trigeminovascular A-fiber responses 2. Effect reversed by OX1R antagonist SB-334867 3. OXB had no significant effect 4. OX1R-mediated modulation involved in migraine-related nociception	Mechanical pain	Holland and Bartsch, (2008)
Acute and chronic pain	Naïve rat	Male wistar rats	Formalin test	1. Intra-LH carbachol reduced formalin-induced biphasic pain 2. VTA OX1R/OX2R blockade weakened LH-induced analgesia 3. Antagonist effects were stronger in the late phase 4. OX1R contributed more than OX2R during late-phase pain	Chemical nociception	Ezzatpanah et al. (2016)
Acute and chronic pain	Naïve rat	Male wistar rats	Formalin test	1. LH stimulation reduced formalin-induced biphasic pain 2. Antinociception observed in both early and late phases 3. OX1R blockade weakened LH-induced antinociception 4. Early-phase response more sensitive to OX1R blockade 5. LH–spinal orexin pathway involved	Chemical nociception	Laleh et al. (2018)
Acute and chronic pain	Naïve rat	Male SD rats	Formalin test	1. OXA in the RVM reduced formalin-induced pain behavior 2. OX1R antagonist blocked orexin A-induced antinociception 3. RVM OX1R signaling involved in chemical pain modulation	Chemical nociception	Hassan et al. (2014)
Acute and chronic pain, pain related behaviors	Naïve mouse	Male C57BL/6	Hot-plate, tail-flick, paw-withdrawal, tail-pressure, formalin, capsaicin, abdominal writhing and hyperalgesia tests	1. Central OXA/OXB produced broad antinociception 2. Effective in thermal, mechanical and chemical pain assays 3. OXA more potent than OXB 4. i.c.v. And i.t. Administration effective; s.c. Administration ineffective	Thermal pain, mechanical pain, chemical nociception, pain-related behavior	Jalal Izadi et al. (2005)

A substantial body of evidence supports a critical role for the orexinergic system in pain modulation. Using both genetic ablation and optogenetic inhibition approaches, Kaneko and colleagues demonstrated that suppression of orexinergic neurons significantly increased capsaicin-induced nociceptive responses following intradermal injection into the cheek, while reducing chloroquine-induced scratching behavior in the neck model of itch (Kaneko et al., 2022). Consistent with these loss-of-function findings, the majority of mechanistic studies have focused on OXA. Central administration of OXA, via both intracerebroventricular (ICV) and intrathecal (IT) routes, produces robust antinociceptive effects, including prolonged thermal withdrawal latency and increased mechanical pain thresholds in assays such as the hot plate test, pressure pain test, and formalin-induced pain models (Jalal Izadi et al., 2005). In neuropathic pain conditions, including chronic constriction injury (CCI) and spared nerve injury (SNI), IT delivery of OXA effectively attenuates mechanical allodynia. A large proportion of preclinical evidence supports a positive association between OXA signaling and antinociception. However, clinical and chronic stress-related findings suggest that elevated orexinergic activity should not be interpreted uniformly as analgesic. Sarchielli and colleagues reported increased OXA levels of cerebrospinal fluid in patients with chronic migraine and medication-overuse headache, and enhanced hypothalamic orexin neuronal activity has also been observed in chronic stress-induced hyperalgesia models (Holland and Bartsch, 2008; Chavan et al., 2022). These observations may reflect nonspecific reactive activation of hypothalamic orexin neurons in response to persistent ascending nociceptive input, or compensatory engagement of descending modulatory systems.

In contrast, the pain-modulatory effects of OXB are less consistent and less extensively characterized. Several studies have directly compared the effects of OXA and OXB on nociceptive processing. In a trigeminovascular pain model, injection of OXB into the posterior hypothalamus increased A- and C-fibre responses to dural stimulation and enhanced spontaneous activity, effects that were opposite to those induced by OXA (Bartsch et al., 2004). Recent evidence further indicates that OXB may promote sex-dependent peripheral nociceptor sensitization. Stratton et al. reported that OXB sensitized dorsal root ganglion neurons from male, but not female, mice, macaques, and humans (Stratton et al., 2024). Nevertheless, other studies suggest that OXB can exert analgesic effects, although with lower potency than OXA. OXB exhibits a higher half-maximal effective concentration (EC50) than OXA reduced potency. Moreover, prolonged administration of OXB has been shown to dose-dependently increase mechanical and thermal withdrawal thresholds in neuropathic pain models, accompanied by reduced spinal expression of pro-inflammatory mediators such as IL-6, TNF-α, and COX-2 (Jalal Izadi et al., 2005; Zhu et al., 2024).

In summary, orexins play a critical role in the modulation of pain. Although OXA is the more extensively studied peptide and often contributes to antinociception through OX1R- and OX2R-related pathways, orexinergic signaling can produce bidirectional effects depending on peptide subtype, receptor distribution, circuit context, sex, and pain modality.

### 2.2 Downstream projection of orexinergic neurons in pain-modulatory circuits

**FIGURE 1:**
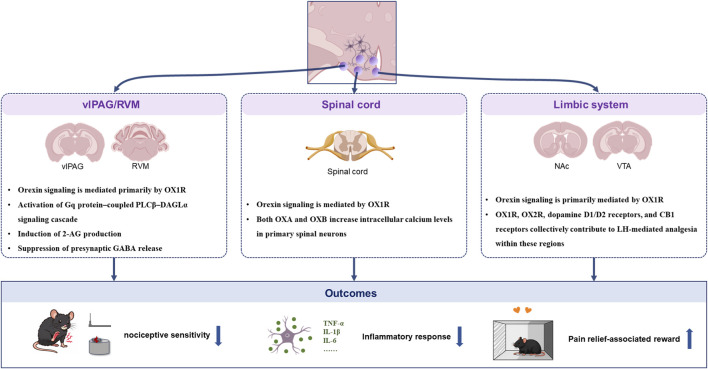
Orexinergic circuit mechanisms underlying pain modulation.

Although orexinergic neurons are anatomically restricted to the LH, they exert widespread influence on pain processing through extensive projections to multiple brain regions and the spinal cord. These projections enable orexinergic neurons to modulate nociception at different hierarchical levels, including descending pain control, spinal transmission, and affective–motivational processing. Accumulating evidence indicates that the analgesic effects of LH orexinergic neurons are mediated not by a single pathway, but by coordinated engagement of multiple projection-defined circuits (Figure 1).

A major mechanism through which orexinergic neurons regulate pain is the activation of the descending pain inhibitory system, particularly via projections to the periaqueductal gray (PAG) and rostral ventromedial medulla (RVM) (Aimone et al., 1988). The PAG, especially its ventrolateral subdivision (vlPAG), is a critical hub in descending pain modulation. Early studies demonstrated that lesioning the PAG abolishes LH-induced increases in pain thresholds, indicating that PAG serves as an essential relay in LH-mediated analgesia. Consistent with this, microinjection of OXA into the PAG significantly prolongs thermal withdrawal latency, supporting a direct antinociceptive role of orexin signaling within this region (Miranda-Páez et al., 2014). More recently, Kaneko et al. showed that LH orexinergic projections to the PAG differentially modulate itch and pain processing, based on optogenetic terminal inhibition experiments (Kaneko et al., 2024). Mechanistically, activation of OX1R in the vlPAG initiates a Gq protein–coupled PLCβ–DAGLα signaling cascade, leading to the production of the endocannabinoid 2-arachidonoylglycerol (2-AG). This retrogradely suppresses presynaptic GABA release via CB1 receptors, resulting in disinhibition of PAG output neurons and activation of descending analgesic pathways (Ho et al., 2011). In parallel, orexin signaling within the RVM has been shown to increase the firing rate of pain-inhibitory neurons, and local OXA administration significantly reduces formalin-induced nocifensive behaviors (Azhdari-Zarmehri et al., 2014). Together, these findings indicate that LH orexinergic neurons exert potent antinociceptive effects through coordinated modulation of the PAG-RVM axis.

In addition to supraspinal circuits, orexinergic neurons also directly influence nociceptive processing at the level of the spinal cord. Pharmacological studies have demonstrated that activation of the LH induces analgesia that can be blocked by intrathecal administration of OX1R antagonists, indicating a critical role for spinal orexin receptors in mediating these effects (Wardach et al., 2016). This pathway appears to be particularly relevant in pathological pain states, as evidenced by studies showing that blockade of spinal OX1R preferentially attenuates the late phase of formalin-induced nociceptive responses (Laleh et al., 2018; Sakineh et al., 2020). Furthermore, the LH-spinal cord pathway has been implicated in mediating the analgesic effects of electroacupuncture, with intrathecal inhibition of OX1R significantly reducing treatment efficacy. Anatomical and *in vitro* studies further support the functional relevance of this projection, revealing dense orexinergic innervation of spinal regions involved in pain processing and demonstrating that both OXA and OXB can elevate intracellular calcium levels in primary spinal neurons (Ozcan et al., 2010; van den Pol, 1999). These findings suggest that orexinergic neurons can exert direct modulatory effects on nociceptive transmission at early stages of the pain pathway.

Beyond sensory-discriminative processing, orexinergic projections to mesolimbic structures contribute to the affective and motivational dimensions of pain. The lateral hypothalamus sends projections to the VTA and nucleus accumbens (NAc), key components of the dopaminergic reward system (Sara et al., 2013). Pharmacological studies indicate that multiple receptor systems, including OX1R, OX2R, dopamine D1 and D2 receptors, and CB1 receptors, participate in LH-mediated analgesia within these regions (Fatemeh et al., 2015; Iman et al., 2018; Marzieh et al., 2015). Functional evidence further demonstrates that activation of the LH^OXA^-NAc pathway induces conditioned place preference for pain relief–associated cues, an effect that is abolished by OX1R antagonism within the NAc (Ezzatpanah et al., 2016). These findings highlight a role for orexin signaling in linking pain relief to reward processing, thereby influencing behavioral responses to pain beyond nociceptive thresholds.

In addition to these major pathways, orexinergic projections to other brain regions, including the locus coeruleus (LC), hippocampus, and additional limbic structures, have also been implicated in pain modulation (Han-Wen et al., 2023). Pharmacological blockade of orexin receptors in the LC and RVM reduces LH-induced analgesia, while receptor antagonism in hippocampal subregions such as CA1 and the dentate gyrus further suggests involvement of cognitive and contextual processing in orexin-mediated pain regulation (Hassan et al., 2014). However, interpretation of these findings is complicated by methodological limitations, including potential diffusion of pharmacological agents and lack of cell type specificity.

Collectively, these findings demonstrate that orexinergic neurons modulate pain through a distributed network of projection-defined circuits spanning the spinal cord, brainstem, and forebrain. Rather than acting through a single pathway, LH orexinergic neurons coordinate multiple functional domains of pain processing, including sensory transmission, descending inhibition, and affective-motivational regulation. This broad integrative capacity positions orexinergic neurons as a central node in the neural control of pain. Future studies combining cell type-specific genetic tools with circuit-level manipulation will be essential to delineate the precise connectivity and functional hierarchy of these pathways, ultimately advancing our understanding of how orexinergic circuits orchestrate pain modulation.

## 3 Cellular heterogeneity and co-transmission in LH pain-modulatory circuits

Although orexinergic neurons have emerged as a central focus in LH-mediated pain regulation, the LH is a highly heterogeneous structure composed of multiple neurochemically and functionally distinct neuronal populations (Mark, 2023). These cell types are anatomically intermingled and, in some cases, exhibit partial co-expression of neuropeptides or classical neurotransmitter markers. Recent single-cell transcriptomic studies have further delineated this heterogeneity, identifying dozens of transcriptionally distinct neuronal subtypes within the LH that display overlapping distributions and potential circuit-level interactions (Mark, 2023).

Partial co-expression of neurotensin and orexin within LH neurons has been reported (Furutani et al., 2013). Neurotensinergic neurons constitute a prominent peptidergic population within the LH. Neurotensin (Nts), originally identified in the hypothalamus, has long been associated with potent antinociceptive effects, as intracerebral administration of Nts or its receptor agonists produces robust analgesia across diverse experimental pain models (Karen et al., 2001). Within the LH, Nts-expressing neurons are preferentially recruited under conditions associated with stress and nociception. For example, stress paradigms such as cold water swim induce a marked increase in Nts mRNA expression in the LH (Geoffroy et al., 2024). Functional studies further demonstrate that selective chemogenetic activation of LH neurotensinergic neurons (LH^Nts^) significantly elevates mechanical pain thresholds in neuropathic and inflammatory pain models, including spared nerve injury (SNI) and CFA-induced inflammation. Notably, these effects appear to be state-dependent, as activation of LH^Nts^ neurons does not alter baseline nociceptive thresholds in naïve animals (Khan et al., 2023; Rabail et al., 2024).

Previous studies have shown that vesicular glutamate transporter 2 (VGLUT2) mRNA labeling is detected in approximately 50% of orexin-immunoreactive neurons, suggesting that glutamatergic signaling partially overlaps with orexinergic neurons in the LH, as VGLUT2 expression is commonly used to identify glutamatergic neurons (Rosin et al., 2003). Evidence from neuropathic pain models indicates that LH^Glu^ neurons are broadly activated under pathological conditions. For instance, increased c-Fos expression has been observed in LH^Glu^ neurons following spared nerve injury, suggesting enhanced excitability in response to chronic pain (Lianghui et al., 2024). In chronic constriction injury models, glutamatergic projections from the LH to the lateral habenula (LH-LHb) exhibit elevated activity, and chemogenetic inhibition of this pathway attenuates nociceptive hypersensitivity, indicating a pronociceptive role for this specific circuit. However, the functional role of LH^Glu^ neurons is not uniform (Han-Wen et al., 2023). A subset of LH neurons co-expressing parvalbumin (PV) and glutamatergic markers forms projections to key pain-modulatory regions, including the ventrolateral periaqueductal gray (vlPAG) and lateral habenula. Optogenetic activation of these LHPV projections to the vlPAG significantly reduces both mechanical and thermal hypersensitivity in inflammatory pain models, whereas distinct LHPV projections to the lateral habenula are more closely associated with aversion-like behavior rather than direct nociceptive modulation (Kisner et al., 2018; Mészár et al., 2012; Siemian et al., 2021). In addition, activation of LHPV neurons produces additive-to-synergistic antinociceptive interactions with morphine and restores morphine antinociception after the development of morphine tolerance (Siemian et al., 2021). These findings highlight a pronounced functional heterogeneity within the LH^Glu^ population, with distinct subcircuits exerting either facilitatory or inhibitory effects on pain depending on their projection targets.

Using immunofluorescent antibody labeling, Muschamp et al. demonstrated that orexin and dynorphin are coexpression in the same neurons and colocalized within the same synaptic vesicles (Muschamp et al., 2014). Dynorphin is widely distributed throughout the brain and, through selective activation of kappa-opioid receptors (KORs), contributes to negative affective states and has been implicated in depressive-like behaviors (Van’t Veer et al., 2013). Previous studies have shown that dynorphin and orexin exert opposing effects on the excitability of VTA dopamine neurons, and that orexin can occlude the reward-threshold-elevating effects of coreleased dynorphin, thereby permissively facilitating reward (Muschamp et al., 2014; Thomas et al., 2022). In addition, LH^GABA^ neurons exerts pro-nociceptive effects that contrast with the role of orexin neurons, through their functional interactions with mesolimbic circuits involved in reward processing and the motivational aspects of pain. Optogenetic activation of the LH^GABA^-VTA projection promotes pain hypersensitivity, whereas inhibition of this pathway produces analgesic effects in CCI-induced neuropathic pain and CFA-induced inflammatory pain (Ma et al., 2023).

## 4 The orexinergic system is a hub for mediating non-pharmacological analgesia

**FIGURE 2:**
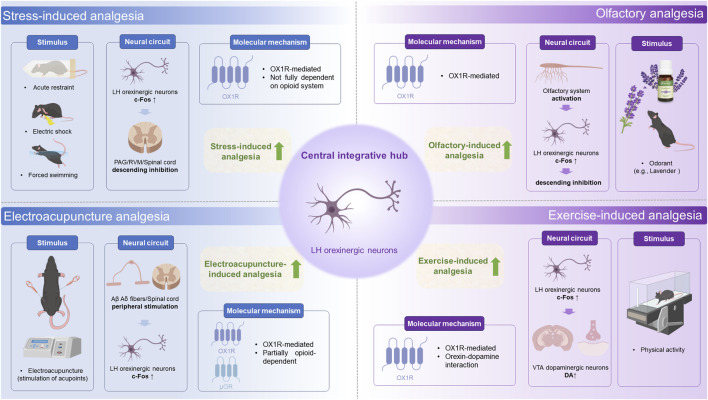
Orexin system as a central hub mediating non-pharmacological analgesia.

In recent years, non-pharmacological interventions have gained considerable attention and been widely incorporated into the comprehensive management of chronic pain. These approaches include, but are not limited to, massage, exercise training, acupuncture, and olfactory stimulation. Most of these strategies are rooted in long-standing cultural practices and accumulated clinical experience, and emphasize the modulation of pain through physical or sensory stimuli rather than pharmacological agents. Despite their heterogeneity, these approaches commonly exert analgesic effects through modulation of central nervous system activity. The orexinergic system serves as a key integrative hub for converging central and peripheral signals and is critically involved in multiple non-pharmacological analgesic mechanisms. Processes including stress-induced analgesia (Dmitry et al., 2011; Nima et al., 2012; Shah et al., 2025; Sofiabad et al., 2011; Watanabe et al., 2005; Xie et al., 2008), olfactory analgesia (Higa et al., 2021; Tashiro et al., 2016), electroacupuncture-induced analgesia (Feng et al., 2012; Ma et al., 2024; Wang et al., 2022), and exercise-induced analgesia (Kami et al., 2018) have all been consistently associated with alterations in orexinergic system activity (Figure 2).

Stress-induced analgesia represents a prototypical form of endogenous pain modulation triggered by acute stress and serves as an adaptive component of the “fight-or-flight” response. Accumulating evidence indicates that the orexinergic system plays a central role in orchestrating this process across multiple levels. Under stress conditions, hypothalamic orexinergic neurons are robustly activated, as evidenced by increased c-Fos expression, and contribute to the suppression of nociceptive transmission via engagement of descending inhibitory pathways. Genetic and pharmacological studies further support this role, as disruption of orexin signaling enhances inflammatory hyperalgesia and attenuates stress-induced analgesia (Shah et al., 2025; Watanabe et al., 2005). At the synaptic level, orexinergic neuronal activity is tightly regulated by interacting neuropeptidergic systems, including nociceptin/orphanin FQ (N/OFQ), which exerts inhibitory control over these neurons and bidirectionally modulates stress-induced analgesia (Dmitry et al., 2011; Xie et al., 2008). This interaction highlights a dynamic balance between excitatory and inhibitory inputs to orexinergic neurons, which determines the net analgesic outcome. Importantly, receptor-specific evidence further indicates that OX1R is a critical mediator of stress-induced analgesia, particularly in models of acute restraint or swim stress, in which blockade of OX1R significantly attenuates stress-induced analgesia without fully engaging opioid pathways (Nima et al., 2012; Sofiabad et al., 2011). It should be noted, however, chronic stress can produce the opposite behavioral outcome, namely, stress-induced hyperalgesia. Chavan et al. revealed that predictable chronic mild stress increases hypothalamic orexin neuronal activity and induces thermal and mechanical hyperalgesia, whereas pharmacological blockade of orexin receptors with suvorexant attenuates these hypersensitivity responses (Chavan et al., 2022). These findings suggest that orexinergic modulation of stress-related pain is stress-duration- and context-dependent, exerting potentially protective antinociceptive effects during acute stress-induced analgesia while contributing to the maintenance of pain hypersensitivity under chronic stress conditions.

Olfactory analgesia represents a distinct form of sensory-driven pain modulation in which odorant stimuli modulate nociceptive processing via central neural pathways (Zhu et al., 2026). Current evidence for orexin-dependent olfactory analgesia is derived mainly from acute nociceptive paradigms rather than chronic pain states. Behavioral studies have demonstrated that exposure to specific odorants, such as linalool, produces robust antinociceptive effects that depend on intact olfactory input, as disruption of the olfactory epithelium or olfactory bulb abolishes these effects. Notably, these odor-induced analgesic responses are accompanied by activation of hypothalamic orexinergic neurons. Genetic or cellular ablation of orexin signaling abolishes this effect, indicating that the orexinergic system is required for the expression of olfactory analgesia (Tashiro et al., 2016). Orexinergic neurons may functionally link olfactory processing regions with descending pain control systems. Activation of orexinergic neurons by odor stimulation is associated with suppression of spinal nociceptive activity. Pharmacological blockade of OX1R prevents odor-induced analgesia and reverses the suppression of spinal c-Fos expression (Higa et al., 2021). These findings support a model in which olfactory inputs recruit hypothalamic orexinergic neurons, which in turn engage descending inhibitory pathways to suppress nociceptive transmission at the spinal level. There is evidence of bidirectional interactions between olfactory perception and the regulatory state of the orexinergic system (Ahasan et al., 2024; Li et al., 2014). However, the upstream mechanisms by which olfactory signals access the orexinergic system remain incompletely defined. Current evidence supports a model in which olfactory stimuli engage hypothalamic orexinergic neurons to initiate descending pain inhibition; however, the precise pathways linking olfactory circuits to the orexinergic system remain to be fully elucidated. Future studies combining circuit tracing and functional manipulation will be required to determine whether this pathway represents a dedicated olfactory-hypothalamic-spinal axis or a broader integrative mechanism shared with other sensory and affective modulators of pain.

In the context of electroacupuncture-induced analgesia, peripheral somatic stimulation is transduced into central signals that engage both spinal and supraspinal mechanisms. Orexin-A signaling, particularly via OX1R, contributes directly to the analgesia, with spinal orexin pathways participating in suppressing nociceptive transmission (Feng et al., 2012). Chen et al. suggested that PC6-targeted low-frequency median nerve stimulation activates hypothalamic orexin neurons, leading to orexin release and analgesia through an OX1R-initiated 2-AG retrograde disinhibition cascade in the vlPAG that depends on CB1R signaling, thereby directly extending the mechanism described by Ho et al. to an acupuncture-related analgesic paradigm (Chen et al., 2018; Ho et al., 2011). Notably, this modulation is not entirely dependent on opioid signaling, suggesting that orexin-mediated electroacupuncture-induced analgesia represents a partially independent endogenous pain control system (Ma et al., 2024). At the same time, electroacupuncture-induced analgesia engages hypothalamic orexin neurons, as evidenced by activity-dependent markers, and extends its effects beyond nociceptive inhibition to modulate affective and motivational components of pain. This is further supported by the involvement of orexin projections to mesolimbic structures, indicating that electroacupuncture-induced analgesia is not solely sensory in nature but also incorporates reward-related processing (Wang et al., 2022).

A similar integrative framework is observed in exercise-induced analgesia, in which physical activity induces analgesia through coordinated activation of hypothalamic and mesolimbic circuits (Kami et al., 2022). Exercise has been shown to enhance the activity of lateral hypothalamic orexin neurons alongside dopaminergic pathways, suggesting that orexin signaling contributes to coupling between reward processing and pain modulation (Kami et al., 2018). This interaction may underlie the sustained and self-reinforcing characteristics of exercise-induced analgesia, whereby the motivational aspects of exercise are linked to its analgesic benefits.

The orexin system plays a pivotal role in essential physiological processes, including sleep-wake regulation, feeding behavior, and cue-based learning, all of which are closely linked to pain perception. Current orexin receptor-targeting drugs have been developed primarily for sleep-wake disorders: orexin receptor antagonists are used for insomnia, whereas orexin receptor agonists are being developed primarily for narcolepsy. A recent systematic review by Babiloni et al. summarized four clinical studies examining the effects of oral dual orexin receptor antagonists on pain-related outcomes, but no consistent analgesic benefit was observed, despite improvements in sleep parameters (Babiloni et al., 2025). These findings do not necessarily contradict the preclinical evidence reviewed above; rather, they suggest that nonselective orexin receptor blockade may not reproduce the analgesic effects produced by activation of specific orexinergic circuits. In particular, many preclinical antinociceptive mechanisms appear to involve activation of OX1R-dependent descending inhibitory pathways. By contrast, emerging OX2R agonists, such as danavorexton and oveporexton, have shown wake-promoting effects and reductions in cataplexy-related symptoms in narcolepsy; however, their clinical development is currently directed toward disorders of wakefulness rather than pain management (Dauvilliers et al., 2025; Dauvilliers et al., 2023). Therefore, future studies should carefully distinguish among receptor subtype, circuit context, pain modality, and behavioral outcome when evaluating the therapeutic potential of orexin-related pathways. Further investigation is warranted to clarify how the orexin system contributes to distinct forms of analgesia and whether selective receptor- or circuit-level modulation can be translated into pain management strategies.

## 5 Conclusion

Pain is the subjective interpretation of noxious stimuli, and its perception does not always correlate linearly with the intensity of nociceptive input. Accumulating evidence demonstrates that lateral hypothalamic orexinergic neurons serves as a central integrative hub in pain regulation. Through widespread projections to the spinal cord, brainstem, and forebrain, these neurons coordinate multiple dimensions of nociceptive processing, affective responses, and pain-related motivational states. OXA is the more extensively studied peptide and often exerts antinociceptive effects through OX1R- and OX2R-related signaling, particularly within descending pain-inhibitory pathways. In contrast, OXB may exert either antinociceptive or pronociceptive effects depending on sex, pain modality, and duration of exposure. Nevertheless, several critical questions remain unsolved. The precise circuit architecture linking sensory inputs, particular olfactory and somatosensory signals, to orexinergic neurons requires further clarification. In addition, because many preclinical studies cited in this review included only one sex or did not perform sex-stratified analyses, the extent to which orexinergic pain modulation differs between males and females remains an important knowledge gap. Overall, the orexinergic system represents a promising target for future research and therapeutic development.
